# Telemonitoring of daily activities compared to the six-minute walk test further completes the puzzle of oximetry-guided interventions

**DOI:** 10.1038/s41598-021-96060-w

**Published:** 2021-08-16

**Authors:** Catarina Duarte Santos, Ana Filipe Santos, Rui César das Neves, Ruy M. Ribeiro, Fátima Rodrigues, Cátia Caneiras, Martijn A. Spruit, Cristina Bárbara

**Affiliations:** 1 Instituto de Saúde Ambiental, Faculdade de Medicina, Universidade de Lisboa, Lisbon, Portugal; 2 Unidade de Reabilitação Respiratória, Hospital Pulido Valente, Centro Hospitalar Universitário Lisboa Norte, Lisbon, Portugal; 3 CAST - Consultoria e Aplicações em Sistemas e Tecnologia, Lda., Lisbon, Portugal; 4 Laboratório de Biomatemática, Instituto de Saúde Ambiental, Faculdade de Medicina da Universidade de Lisboa, Lisbon, Portugal; 5 Laboratório de Microbiologia na Saúde Ambiental (EnviHealthMicroLab), Instituto de Saúde Ambiental, Faculdade de Medicina, Universidade de Lisboa, Lisbon, Portugal; 6 Instituto de Medicina Preventiva e Saúde Pública, Faculdade de Medicina, Universidade de Lisboa, Lisbon, Portugal; 7 Healthcare Department, Nippon Gases, Maia, Portugal; 8 Department of Research and Development, CIRO, 6085 NM Horn, The Netherlands; 9 Department of Respiratory Medicine, NUTRIM School of Nutrition and Translational Research in Metabolism, Faculty of Health, Medicine and Life Sciences, Maastricht University Medical Centre, 6229 HX Maastricht, The Netherlands; 10 Serviço de Pneumologia, Centro Hospitalar Universitário Lisboa Norte, Lisbon, Portugal

**Keywords:** Health care, Medical research, Risk factors, Signs and symptoms

## Abstract

Pulmonary rehabilitation is based on a thorough patient assessment, including peripheral oxygen saturation (SpO_2_) and heart rate (HR) at rest and on exertion. To understand whether exercise-field tests identify patients who desaturate (SpO_2_ < 90%) during physical activities, this study compared the six-minute walk test (6MWT) and daily-life telemonitoring. Cross-sectional study including 100 patients referred for pulmonary rehabilitation. The 6MWT was performed in hospital with continuous assessment of SpO_2_, HR, walked distance and calculated metabolic equivalent of tasks (METs). Patients were also evaluated in real-life by SMARTREAB telemonitoring, a combined oximetry-accelerometery with remote continuous assessment of SpO_2_, HR and METs. SMARTREAB telemonitoring identified 24% more desaturators compared with the 6MWT. Moreover, there were significant mean differences between 6MWT and SMARTREAB in lowest SpO_2_ of 7.2 ± 8.4% (*P* < 0.0005), in peak HR of − 9.3 ± 15.5% (*P* < 0.0005) and also in activity intensity of − 0.3 ± 0.8 METs (*P* < 0.0005). The 6MWT underestimates the proportion of patients with exercise-induced oxygen desaturation compared to real-life telemonitoring. These results help defining oximetry-guided interventions, such as telemedicine algorithms, oxygen therapy titration and regular physical activity assessment in pulmonary rehabilitation.

## Introduction

Pulmonary rehabilitation is based on a thorough patient assessment, which include functional exercise capacity and physical activity^1^. For this purpose it is important to appropriately monitor peripheral oxygen saturation (SpO_2_) and heart rate (HR) at rest and exertion individual responses^2^, as relevant inputs to consider when optimizing oxygen therapy^3–6^ and designing exercise-based interventions^1,7^.

The six-minute walk test (6MWT) is a self-paced exercise-field test with standardized guidelines, assessing walking distance as the primary outcome for functional exercise capacity^8,9^. Furthermore, this test is recommended as more sensitive to detect oxygen desaturation compared to maximal tests^8,10^. Because of this, patients with chronic lung disease should have oxygen desaturation (SpO_2_ < 90%) quantified^11–16^, as there is evidence that desaturation during the test is associated with more severe lung disease, higher levels of dyspnoea, decreased muscle strength, reduced physical activity and daily-life activities desaturation^17^. Moreover, it is also recommended to include measurement of HR in 6MWT assessments, as a mean not only to evaluate HR response, but also to estimate mean exercise intensity as percentage of maximal HR^17^.

Ambulatory pulse oximetry provides an accurate profile on desaturating time^18^, as it reflects the influence of different activities of daily-life such as walking, washing and eating^19^. There is considerable evidence in patients with Chronic Obstructive Pulmonary Disease (COPD) that such method detects hypoxemia with reported desaturation times above 25%^20^, mostly unrevealed by resting arterial blood gases^21^. Furthermore, these patients have daytime oximetry with a higher number of desaturations and lowest detected SpO_2_ (nadir SpO_2_) comparing to nocturnal oximetry with a lower mean SpO_2_^19,22^. A recent systematic review on oximetry of telemonitored patients with COPD, recognized the TELEMOLD technology as unique on continuously measure SpO_2_, instead of doing solely SpO_2_ spot checks^23^. This first telemedicine solution combining oximetry and accelerometery telemonitoring to improve long-term oxygen therapy (LTOT), detected 87% of patients with chronic respiratory failure with hypoxemia and important oxygen desaturation during daily activities^3^. Additionally, the same technology assessing physical activities in daily-life (PADL), detected 93% of patients with chronic respiratory disease with daily oxygen desaturation episodes, and 99.6% of daytime spent on activities with a daily energy expenditure of less than threefold of the basal metabolism^7^.

Considering that physical performance and physical activity are associated but separate domains of physical function^24,25^, it is important to understand if clinical exercise-field tests identify patients who desaturate on PADL. The aim of the present study was to compare the 6MWT of patients with chronic lung disease and PADL telemonitoring outcomes to analyse differences on nadir SpO_2_, peak HR and activity intensity. Our research question was: does the 6MWT identify patients who desaturate on PADL, and are the intensities of such activities comparable to the metabolic demands of the test? Our research hypothesis was that, as a submaximal test, the 6MWT would identify more desaturator patients, although we could not exclude that there could be more intense PADL leading to desaturation comparing to the test, namely those including upper and lower-limb active participation.

## Methods

We implemented a cross-sectional study with a sample of N = 100 patients with chronic lung disease admitted for pulmonary rehabilitation in Hospital Pulido Valente in Lisbon, Portugal. Excluding criteria were: pleural effusion, infectious disease, unstable cardiac disease, neurologic or musculoskeletal conditions affecting exercise performance and cognitive deficit or psychiatric disease. Patients were interviewed about employment status, living environment, smoking status, use of LTOT and non-invasive ventilation. Main condition for pulmonary rehabilitation referral, associated comorbidities and pulmonary function data were obtained by authorized access to the hospital clinical file. All methods were performed in accordance with the relevant guidelines and regulations. All patients gave informed consent and research has been performed in accordance with the Declaration of Helsinki. Ethical approval was obtained from the Ethics Committee of Centro Hospitalar Universitário Lisboa Norte, EPE and Centro Académico de Medicina de Lisboa (number 02/17). Trial was registered as NCT03930511 at clinicaltrials.gov.

### 6MWT

To test functional exercise capacity, patients performed a 6MWT in the hospital, using an indoor straight 10-m hallway, and were given standardised instructions by a physiotherapist^8^. Three tests were performed to overcome the known learning effect^8^, and the 6MWT with the greater distance walked was selected. Patients were continuously monitored for SpO_2_ and HR using a hand-held portable oximeter (BCI 3303 oximeter, Smiths Medical PM, Inc., Waukesha, Wisconsin). Desaturator patients were identified as those with detected SpO_2_ < 90%^11–16^ (primary outcome). Continuous assessment of SpO_2_ during the 6MWT provided secondary outcomes: nadir SpO_2_, number of periods of time with SpO_2_ < 90%, duration of the longest period (minutes) with SpO_2_ < 90%, and the number of pauses during the test. Peak HR was recorded and calculated as a percentage of the theoretical maximal HR using Fox’s age-predicted equation of 220—age. The modified Borg scale was applied for subjective dyspnoea, muscle fatigue and chest pain, pre and post 6MWT; HR and blood pressure were also assessed at those times (Omron BP785 IntelliSense Automatic Blood Pressure Monitor with ComFitTM; Omron Healthcare Co., Ltd; China). Body weight and height were also measured (SECA 220 scale, Seca gmbh & co.kg, Hamburg, Germany). Walking intensity was estimated as METs (metabolic equivalence of task), using the American College of Sports Medicine metabolic prediction equation for the 6MWT: METs = [0.1 × speed (m∙min^−1^) + 3.5 mL O_2_∙Kg∙min^−1^] ÷ 3.5 mL O_2_∙Kg∙min^−1^^26^.

### SMARTREAB telemonitoring

Patients were telemonitored in PADL during 4 consecutive days with remote continuous assessment of SpO_2_, HR and METs of physical activity in daily life. For that purpose, patients were instructed to use, while awake, an Android smartphone with an incorporated accelerometer (Vodafone Smart 4 Turbo, Huawei, China) connected via Bluetooth to a portable transcutaneous oximeter (Avant 4000, Nonin Medical, Plymouth, MN, USA), with finger or ear sensor, as described by the SMARTREAB study^7^. A physiotherapist accessed retrospectively recorded telemonitoring data and crossmatched patient’s qualitative input, describing specificities of carried out tasks (type of activity, duration and perceived exertion). Recorded data analysis identified desaturators with SpO2 < 90%^16,23,27,28^ (primary outcome) and provided secondary outcomes: nadir SpO_2_, number of daily periods with SpO_2_ < 90%, duration of the longest period (minutes) with SpO_2_ < 90%, and also if nadir SpO_2_ was detected within such longest desaturation period. Peak HR was calculated as described above and analysed if it occurred while SpO_2_ < 90%, or even if it was synchronous with nadir SpO_2_ < 90%. Type of activity during nadir SpO_2_ and peak HR were registered, as well as if it had occurred in the morning, afternoon (from 12 am until 5 pm) or evening. Highest activity intensity was recorded as peak MET detected.

### Data analysis

Data management and statistical analysis were performed using the Statistical Package for the Social Sciences (SPSS) version 25.0 (SPSS Inc., Chicago, IL, USA). A formal sample size was not calculated since this was a convenience sample study of 100 chronic lung disease patients. Descriptive statistics of frequencies are presented as percentages for categorical data, and as mean (or median) and standard deviation (or interquartile range) for quantitative variables. Statistical differences between the means in 6MWT and SMARTREAB telemonitoring were assessed with paired-sample t-tests. Mean differences between patients who only desaturated in the SMARTREAB telemonitoring and those who also desaturated in the 6MWT were assessed with independent sample t-tests (except for median differences between number of pauses in the 6MWT and number of periods with SpO_2_ < 90% in the 6MWT and SMARTREAB telemonitoring that were assessed with independent sample Mann–Whitney U tests). A *P*-value of less than 0.05 was considered statistically significant.

### Ethics approval and consent to participate

All patients gave informed consent and ethical approval was obtained from the Ethics Committee of Centro Hospitalar Universitário Lisboa Norte, EPE and Centro Académico de Medicina de Lisboa (number 02/17).

## Results

### Participant characteristics

The recruitment process included 127 people as described in the SMARTREAB study^7^, with 27 not meeting the inclusion criteria. A convenience sample of N = 100 patients was enrolled and the main respiratory diagnoses for pulmonary rehabilitation referral were: COPD (41%), interstitial lung disease (ILD) (22%, mostly idiopathic pulmonary fibrosis), asthma (15%), bronchiectasis (10%) and other (12%, including post-thoracic surgery, lung cancer, tuberculosis sequelae, lung disorder associated with connective tissue disease and pulmonary ossification). Patients had a mean age of 66.1 ± 9.8 years, 50% where male and anthropometry data, pulmonary function, smoking status, use of LTOT and non-invasive ventilation are presented in Table 1.

**Table 1:** Patients’ characteristics per chronic lung disease.

	Total	COPD	ILD	Asthma	Bronch	Other
	N = 100	N = 41	N = 22	N = 15	N = 10	N = 12
Male, n (%)	50 (50.0)	31 (75.6)	8 (36.4)	4 (26.7)	3 (30.0)	4 (33.3)
Age (years; mean ± SD)	66.1 ± 9.8	68.0 ± 7.2	63.5 ± 9.5	66.0 ± 11.9	68.0 ± 13.2	63.2 ± 11.9
**Anthropometry**						
Weight (Kg; mean ± SD)	69.7 ± 15.0	71.4 ± 14.5	70.0 ± 15.9	72.0 ± 14.3	63.4 ± 12.7	65.5 ± 17.8
Height (m; mean ± SD)	1.62 ± 0.1	1.66 ± 0.1	1.59 ± 0.1	1.56 ± 0.1	1.60 ± 0.1	1.62 ± 0.1
BMI (Kg/m^2^; mean ± SD)	26.5 ± 5.1	25.8 ± 5.0	27.7 ± 5.6	29.4 ± 5.0	24.5 ± 4.0	24.7 ± 4.2
**Pulmonary function**						
FEV_1_ pred (%; mean ± SD)	62.1 ± 27.2	53.2 ± 25.8	71.3 ± 20.0	78.6 ± 31.0	69.6 ± 35.7	63.3 ± 23.4
FVC pred (%; mean ± SD)	80.2 ± 25.5	86.5 ± 25.0	69.7 ± 18.0	94.1 ± 26.3	84.4 ± 23.0	84.5 ± 13.8
FEV_1_/FVC (%; mean ± SD)	73.3 ± 25.1	57.3 ± 20.3	101.4 ± 19.6	77.5 ± 19.1	70.8 ± 19.2	69.8 ± 24.1
TLC pred (%; mean ± SD)	102.5 ± 27.6	117.3 ± 21.7	73.0 ± 22.4	108.3 ± 22.8	110.3 ± 14.8	107.2 ± 26.6
RV pred (%; mean ± SD)	142.1 ± 63.9	173.4 ± 65.2	82.8 ± 58.2	134.8 ± 36.0	158.7 ± 31.0	147.2 ± 63.1
DLCO pred (%; mean ± SD)	57.0 ± 24.4	52.1 ± 23.9	47.2 ± 18.5	80.0 ± 13.6	65.3 ± 35.8	61.1 ± 12.7
**Smoking status**						
Non-smoker, n (%)	40 (40.0)	2 (4.9)	11 (50.0)	9 (60.0)	10 (100)	8 (66.7)
Former smoker, n (%)	52 (52.0)	34 (82.9)	10 (45.5)	4 (26.7)	0 (0.0)	4 (33.3)
Active smoker, n (%)	8 (8.0)	5 (12.2)	1 (4.5)	2 (13.3)	0 (0.0)	0 (0.0)
LTOT, n (%)	29 (29.0)	16 (39.0)	11 (50.0)	0 (0.0)	1 (10.0)	1 (8.3)
Nocturnal NIV, n (%)	12 (12.0)	5 (12.2)	3 (13.6)	3 (20.0)	1 (10.0)	0 (0.0)

COPD: chronic obstructive pulmonary disease; ILD: interstitial lung disease; Bronch.: bronchiectasis; Other: post-thoracic surgery, lung cancer, tuberculosis sequelae, lung disorder associated with connective tissue disease and pulmonary ossification; SD: standard deviation; BMI: body mass index; FEV_1_ pred: predicted forced expiratory volume in 1 s; FVC pred: predicted forced vital capacity; FEV_1_/FVC: forced expiratory volume in 1 s and forced vital capacity ratio; TLC pred: predicted total lung capacity; RV pred: predicted residual volume; DLCO pred: predicted diffusion capacity of the lungs for carbon monoxide; LTOT: long-term oxygen therapy; NIV: non-invasive ventilation.

A total of 22 different comorbidities were reported, with a median of 5 per patient of which the most frequent were: arterial hypertension (56%), dyslipidaemia (40%) and anxiety or depression (29%). Detailed information about comorbidities per chronic lung disease are provided as Supplementary Information.

### 6MWT

Patients walked a mean distance of 328.1 ± 113.9 m in the six minutes with 67% of patients desaturating during the test. There was no pause during the 6MWT for 81% of the patients, 14% had 1 pause, and 5% paused at least twice. Continuous oximetry revealed that 45% of patients had one single bout of time with SpO_2_ < 90% during the test, 18% had two periods of desaturation and 4% of the patients had three desaturation periods. We also found that the mean longest period during the 6MWT with SpO_2_ < 90% was of 2.1 ± 2.1 min. Our results also showed that 50% of patients desaturated between the 1^st^ and 3^rd^ minute of the 6MWT. Through the modified Borg scale, dyspnea had a median (interquartile range) of 3 (2–4), and muscle fatigue had a median (interquartile range) of 0.5 (0–3). There was no reported chest pain for 80% of the patients. All 29 patients already under long-term oxygen therapy performed the test carrying their own equipment and complied with the oxygen debit as formerly prescribed by the patient’s physician. Detailed 6MWT information per chronic lung disease is provided as Supplementary Information.

### SMARTREAB telemonitoring

**Figure 1:**
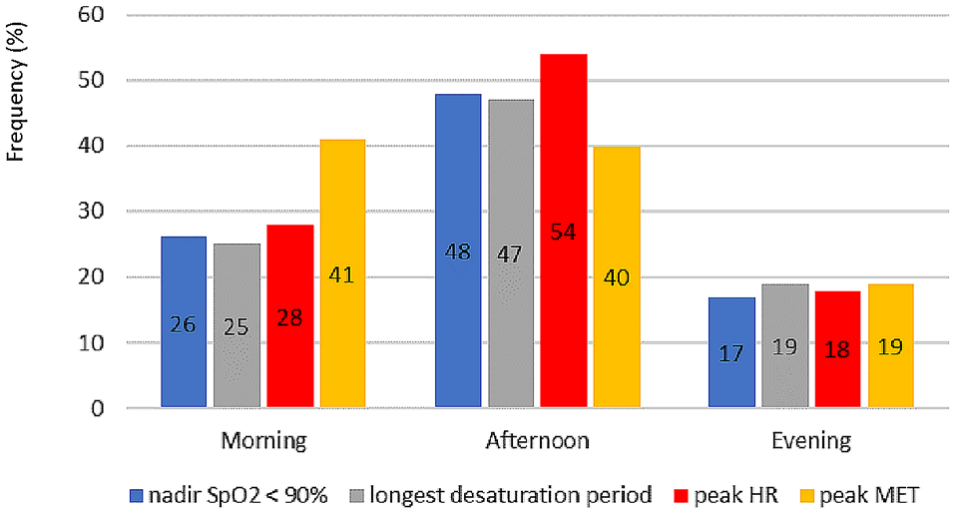
Distribution of the frequency of nadir SpO_2_ < 90%, longest desaturation period, peak HR and peak MET daily-life activities evaluated by SMARTREAB telemonitoring for different periods of the day (morning, afternoon and evening).

During the telemonitoring period, 91% of patients desaturated during PADL, with a median (interquartile range) of 6.5 (2.0–11.8) periods per day with SpO_2_ < 90%. The longest desaturation period of the day had a median (interquartile) duration of 14 (3–37) minutes. As illustrated in Fig. 1, peak MET activities frequency was similar in the morning and in the afternoon, but patients reached peak HR, lowest levels of SpO_2_ and suffered longest periods of oxygen desaturation mostly in the afternoon.

**Figure 2:**
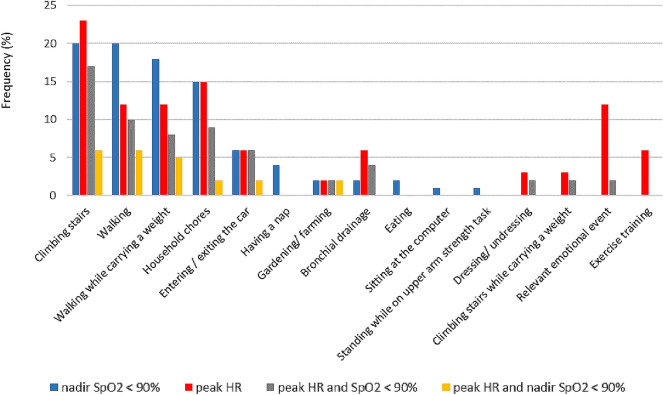
Frequency of daily-life activities, nadir SpO_2_ < 90%, peak HR, peak HR with desaturation and peak HR synchronous with nadir SpO_2_ < 90% as evaluated by SMARTREAB telemonitoring. Activities are ordered (left to right) from highest frequency of desaturating events.

Considering patients reported PADL cross-matched with telemonitoring data, Fig. 2 presents the frequency of activities with nadir SpO_2_ < 90% and peak HR detected by SMARTREAB telemonitoring.

Overall, there were 91% of patients engaging desaturating PADL, 62% reaching their peak HR with desaturation (17% climbing stairs, 10% walking and 9% household chores) and 23% with synchronous peak HR and nadir SpO_2_ < 90% (6% climbing stairs and walking and 5% walking while carrying a weight). Detailed SMARTREAB telemonitoring information per chronic lung disease is provided as Supplementary Information.

### 6MWT vs SMARTREAB telemonitoring

SMARTREAB telemonitoring detected that 91% of patients engaged on desaturating PADL, whereas the 6MWT only identified 67% of patients with nadir SpO_2_ < 90% during the exercise-field test (primary outcome). We found statistically significant mean differences in nadir SpO_2_, peak HR and activity intensity performed between both methods of assessment, as presented in Table 2.

**Table 2:** 6MWT and SMARTREAB telemonitoring compared.

	6MWT	SMARTREAB	Difference of means*	95% CI	*P*-value**
Nadir SpO_2_ (%; mean ± SD)	86.2 ± 6.2	79.0 ± 8.2	7.2 ± 8.4	5.6 to 8.9	< 0.0005
Peak HR (%; mean ± SD)	71.2 ± 11.9	80.6 ± 11.2	− 9.3 ± 15.5	− 12.4 to − 6.2	< 0.0005
Activity intensity (MET; mean ± SD)	2.6 ± 0.5	2.9 ± 0.7	− 0.3 ± 0.8	− 0.5 to − 0.2	< 0.0005

*Calculated pairwise.

**Paired sample t-test.

Nadir SpO_2_: lowest peripheral oxygen saturation detected; HR: heart rate; MET: metabolic equivalent of task; 6MWT: six-minute walk test; CI: confidence interval; SD: standard deviation.

The SMARTREAB telemonitoring detected lower nadir SpO_2_ and higher peak HR for more intense PADL compared with the 6MWT. Regarding the 29% of patients under oxygen therapy, all patients desaturated in real-life SMARTREAB telemonitoring and only 2 patients did not desaturate in the 6MWT.

**Figure 3:**
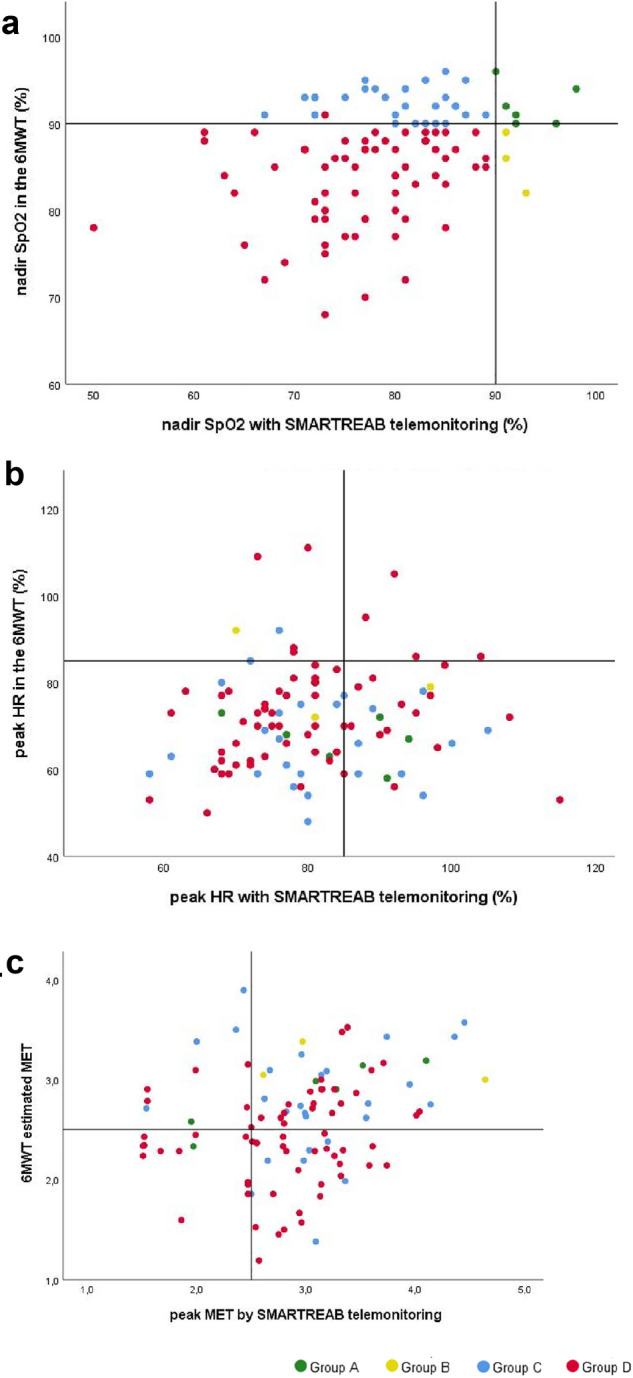
Six-minute walk test and SMARTREAB telemonitoring compared: (**a**) lowest peripheral oxygen saturation detected (nadir SpO_2_), with quadrant line references of 90% nadir SpO_2_; (**b**) peak heart rate (peak HR), with quadrant line references of 85% peak HR; and (**c**) activity intensity by metabolic equivalent of task (MET), with quadrant line references of 2.5 MET. All scatter graphs indicate distribution of non-desaturator patients (group A in green; N = 6), only 6MWT desaturator patients (group B in yellow; N = 3); only SMARTREAB telemonitoring desaturators (group C in blue; N = 27) and both methods desaturators (group D in red; N = 64).

Overall, results demonstrated that 64% of patients desaturated on both assessments whereas 6% of patients on neither of the two. We also found that 30% of patients were identified as desaturators by one method only: 3% in the 6MWT and 27% with the SMARTREAB telemonitoring. This is illustrated in Fig. 3a which compares both methods on detected nadir SpO_2_, highlighting non-desaturators (group A in green), only 6MWT desaturators (group B in yellow), only SMARTREAB desaturators (group C in blue) and both methods desaturators (group D in red). Figure 3b compares the same groups on peak HR, and it shows that the 85% peak HR was surpassed by 11% of patients in the 6MWT and 32% of patients on PADL. Figure 3c compares again the same groups but on activity intensity, and it shows that 2.5 METs intensity was surpassed by 56% of the patients in the 6MWT, and 77% of the patients in real-life SMARTREAB telemonitoring.

To understand why there were patients only desaturating in the SMARTREAB telemonitoring but not in the 6MWT, we compared mean differences between groups C and D as presented in Table 3.

**Table 3:** Only SMARTREAB desaturators and both desaturators compared.

	Group C	Group D	Difference of means or medians*	95% CI	*P*-value
	N = 27	N = 64			
Age (years; mean ± SD)	62.8 ± 12.0	67.7 ± 8.1	− 4.8 ± 2.2	− 9.1 to − 0.5	0.028**
**Pulmonary function**					
FVC pred (%; mean ± SD)	94.7 ± 19.9	73.7 ± 25.4	21.1 ± 5.0	11.1 to 31.0	< 0.0005**
DLCO pred (%; mean ± SD)	71.0 ± 23.8	49.7 ± 22.2	21.2 ± 5.9	9.2 to 33.2	0.001**
**6MWT**					
6MWD (m; mean ± SD)	375.6 ± 121.6	296.5 ± 103.8	79.1 ± 26.8	25.1 to 133.0	0.005**
Nadir SpO_2_ (%; mean ± SD)	92.4 ± 1.8	83.1 ± 5.4	9.3 ± 1.1	7.2 to 11.4	< 0.0005**
Peak HR (%; mean ± SD)	67.2 ± 10.6	72.9 ± 12.4	− 5.7 ± 2.6	− 10.8 to − 0.6	0.030**
Activity intensity (MET; mean ± SD)	2.8 ± 0.6	2.4 ± 0.5	0.4 ± 0.1	0.1 to 0.6	0.005**
Number of pauses (*n*; median, IQR)	0 (0–0)	0 (0–1)	− 2.5	–	0.016***
Number of periods SpO_2_ < 90% (*n*; median, IQR)	0 (0–0)	1 (1–2)	− 8.1	–	< 0.0005***
**SMARTREAB telemonitoring**					
Nadir SpO_2_ (%; mean ± SD)	80.3 ± 5.8	76.5 ± 7.6	3.8 ± 1.5	0.9 to 6.7	0.011**
Peak HR (%; mean ± SD)	80.5 ± 11.3	80.2 ± 11.4	0.3 ± 2.6	− 4.9 to 5.5	0.905**
Activity intensity (MET; mean ± SD)	3.1 ± 0.7	2.8 ± 0.6	0.3 ± 0.2	0.0 to 0.6	0.069**
Daily episodes SpO_2_ < 90% (*n*; median, IQR)	2 (1–5)	9 (5–17)	− 4.3	–	< 0.0005***
Longest period SpO_2_ < 90% (min; mean ± SD)	25.4 ± 41.8	32.8 ± 35.4	− 7.4 ± 9.2	− 26.0 to 11.1	0.424**

*Means and medians calculated independently.

**Independent sample t-test.

***Independent sample Mann–Whitney U test.

Group C: only SMARTREAB telemonitoring desaturators; Group D: six-minute walk test and SMARTREAB telemonitoring desaturators; CI: confidence interval; SD: standard deviation; IQR: interquartile range; FVC pred: predicted forced vital capacity; DLCO pred: predicted diffusion capacity of the lungs for carbon monoxide; 6MWT: six-minute walk test; 6MWD: six-minute walk distance; nadir SpO_2_: lowest peripheral oxygen saturation detected; HR: heart rate; MET: metabolic equivalent of task.

Patients who desaturated on both assessment methods are older and have lower forced vital capacity, as well as lower diffusion capacity of the lungs. This group represents 75.6% of COPD and 72.7% of ILD patients within the overall studied sample. While patients from group C had no desaturation in the 6MWT, group D patients not only desaturated, but also walked shorter distances reaching higher peak HR, with pauses and with more than 1 desaturating period during the test. Although there were no significant differences between group C and D patients in their PADL intensity or peak HR, group D patients had the lowest nadir SpO_2_ and a median of more 4 daily desaturating episodes (without a significant difference between the longest desaturation period when comparing both groups).

## Discussion

The main finding of this study was that ambulatory telemonitoring, namely SMARTREAB telemonitoring, when compared with the 6MWT, identified 24% more desaturators, detecting lower nadir SpO_2_ and higher peak HR for more intense PADL, with statistical significance.

Our results show a higher sensitivity of daily-life telemonitoring compared to the 6MWT in detecting patients with chronic lung disease who desaturate, as 91% engaged on desaturating PADL and only 67% desaturated in the 6MWT. More thoroughly, only 64% of daily desaturators were also 6MWT desaturators, which is in accordance with previous evidence^29^. Furthermore, the García-Talavera group found that COPD patients who desaturated within the 1st minute of the 6MWT also desaturated in the 24-h oximetry, whereas those that only desaturated after 3min30s of the 6MWT did not desaturate in the 24-h oximetry^30^. With a 4-day telemonitoring assessment, we found that 50% of patients who are daily desaturators also desaturate between the 1st and 3rd minute of the 6MWT. Because of this we recommend that patients who desaturate on the first half of the 6MWT should be further assessed by an ambulatory pulse oximetry. Furthermore, we must also consider that PADL is a complex behaviour, encompassing all forms of activity performed by everyone, with little regard to physical fitness and often structured with conservation of energy expenditure as a goal^24,31^. This has been demonstrated in patients with COPD by Koolen, when cross-matching the distance in the 6MWT with the number of steps per day, revealing four distinctive patterns: 34% “can’t do, don’t do”; 31% “can do, do do”; 21% “can’t do, do do” and 14% “can do, don’t do”^25^. Our study found that 27% of patients desaturated in PADL but performed a 6MWT with SpO_2_ ≥ 90%. Such finding calls into question the appropriateness of the expression “can do”, as it was based on distance walked as quantity (meters) regardless of quality (oxygen saturation), and related implications in daily-life. Our analyses revealed that these daily desaturators with SpO_2_ ≥ 90% in the 6MWT were younger and had less severe chronic lung disease, and there were significant differences in age, forced vital capacity, lung-diffusion capacity, daily number of desaturating episodes and nadir SpO_2_ detected by SMARTREAB telemonitoring.

There are studies simulating PADL in laboratory tests designed to measure oxygen desaturation^32–34^. For example, a recent publication comparing the 6MWT with a 20-min walking circuit^35^ found that the 6MWT detected higher nadir SpO_2_ and lower peak HR, but there were no results comparing activity intensity as energy expenditure between tests^35^. With SMARTREAB telemonitoring, our study further contributes with the findings that lower nadir SpO_2_ and higher peak HR are detected in more intensive PADL compared to the 6MWT, such as walking, walking while carrying a weight, climbing stairs and doing household chores. In fact, the 4-day telemonitoring period, compared to the 6MWT, improves clinical decision by evaluating patients’ real-life situations with a synchronized assessment of SpO_2_, HR and METs. This enabled the finding that 62% of patients reached peak HR with desaturation and 23% of patients had synchronous peak HR and nadir SpO_2_ < 90%, on PADL with a mean of 2.9 ± 0.7 peak METs, larger than the estimated 6MWT mean of 2.6 ± 0.5 METs.

The fact that the 6MWT was not performed on the recommended 30 m course length may be considered a limitation of the protocol, but due to unavailability of hospital facilities for such purpose, the 6MWT was performed on a 10 m hallway, in accordance with previous published evidence^36^.

### Implications to oximetry guided interventions

Ambulatory telemonitoring, namely SMARTREAB telemonitoring, further detected a median of 2 to 9 daily episodes with SpO_2_ < 90% and longest period of desaturation of 25 to 33 min per patient desaturator. Such results increase evidence that intraday SpO_2_ fluctuations have to be taken into account when algorithms of telemedicine assistance are determined by telemonitoring applications merely based on a daily SpO_2_ spot check. A breakthrough example is the EDGE (sElf-management anD support proGrammE) project, where SpO_2_ is the most predictive vital sign to monitor^37^, applied in a COPD exacerbation prediction algorithm which considers intra-patient SpO_2_ variability over time^38^. With SMARTREAB telemonitoring we found a «win–win» situation, aligned with the recognition that patients perceive significant potential for wearables and apps to help manage their condition^39,40^.

The higher accuracy of SMARTREAB telemonitoring, compared to the 6MWT, is also of interest for clinicians who report lack of resources to correctly and efficiently prescribe oxygen systems^4,41^. Even though arterial blood gases are the first-line diagnostic tool to determine the eligibility for oxygen therapy^5,6^, a single analysis may not be reliable for the adequacy of LTOT^4,42^. Aligned with the evidence that patients with COPD in LTOT normoxic while awake and at rest have 30% diurnal desaturation time and a nadir SpO_2_ < 60%^43^, our results demonstrated that 27% of patients do not desaturate on a 6MWT but are daily desaturators. While research exploring methods of oxygen flow titration with exercise is under development^44–47^, these results should leverage the ambulatory oximetry preciseness to be applied among clinicians for oxygen prescription purposes.

Finally, even though physical activity measurement has been mainly regarded as an outcome of pulmonary rehabilitation^1,48^, this study further extends its relevance as a baseline vital sign and as an input on individually-tailored rehabilitation goals^7^. Recent research identified inactive, active improvers and active decliners as 3 distinct patterns on patients with COPD physical activity natural progression over 1-year^49^. Not only efficiently targeted intervention on PADL is needed to promote long sustainable healthy habits, as there is growing evidence that its systematic assessment might be more sensitive detecting progressive disease deterioration^50^. Because of this, we recommend a comprehensive regular assessment with oximetry and accelerometery, as it provides combined perspectives about PADL: quantity (duration), intensity (METs), modality (type) and quality (SpO_2_ and HR).

## Conclusions

This study found that the 6MWT underestimates the proportion of patients with exercise-induced oxygen desaturation compared to real-life telemonitoring. SMARTREAB telemonitoring, when compared to the 6MWT identified 24% more desaturators, detecting lower nadir SpO_2_ and higher peak HR for more intense PADL. Such results help defining clinical oximetry-guided interventions, such as telemedicine algorithms, oxygen therapy titration and regular physical activity assessment in pulmonary rehabilitation.

## Supplementary Information

Supplementary Information.

## Data Availability

All data generated or analysed during this study are included in this published article and its supplementary information files.

## Abbreviations

6MWT: Six-minute walk test
COPD: Chronic obstructive pulmonary disease
HR: Heart rate
ILD: Interstitial lung disease
LTOT: Long-term oxygen therapy
METs: Metabolic equivalence of tasks
nadir SpO_2_: Lowest detected peripheral oxygen saturation
PADL: Physical activities of daily-life
SpO_2_: Peripheral oxygen saturation
