# Supplementary material for: Telemonitoring of daily activities compared to the six-minute walk test further completes the puzzle of oximetry-guided interventions
Source: Sci Rep. 2021 Aug 16;11:16600. doi: 10.1038/s41598-021-96060-w (PMC8367992; doi:10.1038/s41598-021-96060-w)
Supplement: Supplementary file 1 — Supplementary Information. [file 41598_2021_96060_MOESM1_ESM.pdf]

Telemonitoring of daily activities compared to the six-minute walk test further completes the puzzle of oximetry-guided interventions

Santos CD, Santos AF, das Neves RC, Ribeiro RM, Rodrigues F, Caneiras C, Spruit MA and Bárbara C

Catarina Duarte Santos, MSc * fisiocsantos@gmail.com

Instituto de Saúde Ambiental, Faculdade de Medicina, Universidade de Lisboa, Portugal

Unidade de Reabilitação Respiratória, Hospital Pulido Valente, Centro Hospitalar Universitário Lisboa Norte, Portugal

Ana Filipe Santos, Dr.

Unidade de Reabilitação Respiratória, Hospital Pulido Valente, Centro Hospitalar Universitário Lisboa Norte, Portugal

Rui César das Neves, Dr.

CAST - Consultoria e Aplicações em Sistemas e Tecnologia, Lda., Portugal

Ruy M. Ribeiro, PhD

Laboratório de Biomatemática, Instituto de Saúde Ambiental, Faculdade de Medicina da Universidade de Lisboa, Portugal

Fátima Rodrigues, PhD

Instituto de Saúde Ambiental, Faculdade de Medicina, Universidade de Lisboa, Portugal

Unidade de Reabilitação Respiratória, Hospital Pulido Valente, Centro Hospitalar Universitário Lisboa Norte, Portugal

Cátia Caneiras, PhD

Laboratório de Microbiologia na Saúde Ambiental (EnviHealthMicroLab), Instituto de Saúde Ambiental, Faculdade de Medicina, Universidade de Lisboa, Portugal

Instituto de Medicina Preventiva e Saúde Pública, Faculdade de Medicina, Universidade de Lisboa, Portugal

Healthcare Department, Nippon Gases Portugal

Martijn A. Spruit, PhD

Department of Research & Development, CIRO, 6085 NM Horn, The Netherlands

Department of Respiratory Medicine, Maastricht University Medical Centre, Faculty of Health, Medicine and Life Sciences, NUTRIM School of Nutrition and Translational Research in Metabolism, 6229 HX Maastricht, The Netherlands

Cristina Bárbara, PhD

Instituto de Saúde Ambiental, Faculdade de Medicina, Universidade de Lisboa, Portugal

Serviço de Pneumologia, Centro Hospitalar Universitário Lisboa Norte, Portugal

Table S1 – Comorbidities per chronic lung disease

	Total<br>N = 100<br>%	COPD<br>N = 41<br>*n* (%)	ILD<br>N = 22<br>*n* (%)	Asthma<br>N = 15<br>*n* (%)	Bronchiectasis<br>N = 10<br>*n* (%)	Other<br>N =12<br>*n* (%)
**Respiratory Comorbidities**						
Bronchiectasis	28.0	14 (34.1)	3 (13.6)	8 (53.3)	0 (0.0)	3 (25.0)
Chronic Respiratory Failure	26.0	13 (31.7)	6 (27.3)	1 (6.7)	2 (20.0)	4 (33.3)
Pulmonary Fibrosis	20.0	1 (2.4)	17 (77.3)	1 (6.7)	0 (0.0)	1 (8.3)
Rhinitis	13.0	4 (9.8)	2 (9.1)	3 (20.0)	1 (10.0)	3 (25.0)
Tuberculosis Sequelae	12.0	5 (12.2)	0 (0.0)	1 (6.7)	5 (50.0)	1 (8.3)
Sleep Apnea	9.0	4 (9.8)	2 (9.1)	1 (6.7)	0 (0.0)	2 (16.7)
Lung Cancer	7.0	3 (7.3)	0 (0.0)	0 (0.0)	0 (0.0)	4 (33.3)
Pulmonary Hypertension	7.0	4 (9.8)	0 (0.0)	1 (6.7)	0 (0.0)	2 (16.7)
**Cardiovascular Comorbidities**						
Arterial Hypertension	56.0	23 (56.1)	7 (31.8)	11 (73.3)	8 (80.0)	7 (58.3)
Dyslipidaemia	40.0	17 (41.5)	11 (50.0)	5 (33.3)	3 (30.0)	4 (33.3)
Arrhythmias	11.0	6 (14.6)	3 (13.6)	2 (13.3)	0 (0.0)	0 (0.0)
Coronary Heart Disease	9.0	4 (9.8)	2 (9.1)	2 (13.3)	1 (10.0)	0 (0.0)
Peripheral Arterial Disease	9.0	5 (12.2)	2 (9.1)	2 (13.3)	0 (0.0)	0 (0.0)
**Other Comorbidities**						
Anxiety or Depression	29.0	11 (26.8)	8 (36.4)	6 (40.0)	2 (20.0)	2 (16.7)
Gastric Disease	19.0	5 (12.2)	6 (27.3)	1 (6.7)	4 (40.0)	3 (25.0)
Diabetic	18.0	6 (14.6)	7 (31.8)	3 (20.0)	2 (20.0)	0 (0.0)
Gastroesophageal Reflux Disease	15.0	1 (2.4)	6 (27.3)	4 (26.7)	1 (10.0)	3 (25.0)
Obesity	12.0	5 (12.2)	4 (18.2)	2 (13.3)	1 (10.0)	0 (0.0)
Rheumatoid Disease	11.0	4 (9.8)	4 (18.2)	1 (6.7)	1 (10.0)	1 (8.3)
Thyroid Disease	10.0	2 (4.9)	2 (9.1)	2 (13.3)	2 (20.0)	2 (16.7)
Osteoporosis	7.0	1 (2.4)	3 (13.6)	1 (6.7)	0 (0.0)	2 (16.7)

COPD: Chronic Obstructive Pulmonary Disease; ILD: Interstitial Lung Disease; Other: post-thoracic surgery status, Lung Cancer, Tuberculosis sequelae, Connective Tissue Disease and Pulmonary Ossification

Table S2 – Six-minute walk test outcomes per chronic lung disease

	**Total**<br>N = 100	**COPD**<br>N = 41	**ILD**<br>N = 22	**Asthma**<br>N = 15	**Bronchiectasis**<br>N = 10	**Other**<br>N = 12
6MWT						
6MWD (m; mean ± SD)	328 ± 114	330 ± 127	291 ± 104	341 ± 112	311 ± 88	389 ± 91
nadir SpO_2 (%; mean ± SD)	86.2 ± 6.2	85.4 ± 5.7	83.3 ± 7.4	89.4 ± 3.5	88.6 ± 7.5	88.2 ± 4.9
peak HR (%; mean ± SD)	71.2 ± 11.9	74.3 ± 11.1	69.4 ± 11.4	69.1 ± 12.2	72.5 ± 18.1	65.8 ± 6.5
activity intensity (MET; mean ± SD)	2.6 ± 0.5	2.6 ± 0.6	2.4 ± 0.5	2.6 ± 0.5	2.5 ± 0.4	2.9 ± 0.4
number of pauses (*n*; median; IQR)	0 (0 – 0)	0 (0 -0)	0 (0 – 1)	0 (0 – 0)	0 (0 – 0)	0 (0 – 0)
number of periods SpO_2 < 90% (*n*; median; IQR)	1 (0 – 1)	1 (1 – 1)	1 (0 – 2)	1 (0 – 1)	0 (0 – 1)	1 (0 – 2)

COPD: Chronic Obstructive Pulmonary Disease; ILD: Interstitial Lung Disease; Other: post-thoracic surgery status, Lung Cancer, Tuberculosis sequelae, Connective Tissue Disease and Pulmonary Ossification; 6MWT: six-minute walk test; 6MWD: six-minute walk distance; SD: standard-deviation; nadir SpO_2: lowest peripheral oxygen saturation detected; HR: heart rate; MET: metabolic equivalent of task; IQR: interquartile range

Table S3 – SMARTREAB telemonitoring outcomes per chronic lung disease

	Total<br><br>N = 100	COPD<br><br>N = 41	ILD<br><br>N = 22	Asthma<br><br>N = 15	Bronchiectasis<br><br>N = 10	Other<br><br>N =12
SMARTREAB telemonitoring						
nadir SpO_2 (%; mean ± SD)	80.0 ± 8.2	81 ± 8	75 ± 8	77 ± 9	79 ± 6	82 ± 8
peak HR (%; mean ± SD)	80.6 ± 11.2	80.3 ± 12.0	78.0 ± 10.4	82.6 ± 10.7	84.0 ± 11.5	80.7 ± 10.4
activity intensity (MET; mean ± SD)	2.9 ± 0.7	2.9 ± 0.7	2.9 ± 0.7	2.9 ± 0.4	2.6 ± 0.6	3.5 ± 0.8
daily episodes SpO_2 < 90% (*n*; median; IQR)	6 (2 – 6)	8 (2 – 12)	10 (4 – 21)	3 (1 – 7)	4 (2 – 8)	2 (0 – 15)
longest period SpO_2 < 90% (min; mean ± SD)	28 ± 37	28 ± 36	33 ± 28	26 ± 45	19 ± 32	29 ± 49

COPD: Chronic Obstructive Pulmonary Disease; ILD: Interstitial Lung Disease; Other: post-thoracic surgery status, Lung Cancer, Tuberculosis sequelae, Connective Tissue Disease and Pulmonary Ossification; nadir SpO_2: lowest peripheral oxygen saturation detected; SD: standard-deviation; HR: heart rate; MET: metabolic equivalent of task; IQR: interquartile range
